# Reforming research assessment

**DOI:** 10.7554/eLife.00855

**Published:** 2013-05-16

**Authors:** Randy Schekman, Mark Patterson

**Keywords:** research assessment, science policy, scientific publishing, publishing, eLife

## Abstract

It is time for the research community to rethink how the outputs of scientific research are evaluated and, as the San Francisco Declaration on Research Assessment makes clear, this should involve replacing the journal impact factor with a broad range of more meaningful approaches.

One of the aims of *eLife* is to publish research articles in all areas of the life sciences and biomedicine, ranging from insights into basic biology through to translational and more applied work, and to date we have published articles on topics ranging from genome editing and plant-predator interactions to global life expectancy and the neurobiology of walking.

The impacts of such a broad range of research topics will be similarly diverse. Some articles will stimulate further research by other scientists in the same field, some will lead to clinical or commercial applications, some will be covered in the media and be of interest to the public, some will achieve all of the above and some, inevitably, will have limited impact. The recently released San Francisco Declaration on Research Assessment (http://www.ascb.org/SFdeclaration.html) aims to ‘improve the ways in which the output of scientific research is evaluated by funding agencies, academic institutions, and other parties’.

Currently, however, there is a widespread perception that research assessment is dominated by a single metric, the journal impact factor, which is the average rate of citation to a given journal over a short period. There are many reasons why the impact factor of a journal cannot and should not be used as a proxy for the importance of individual articles in the journal (Seglen, 1992; Adler et al., 2008; Campbell, 2008; Curry, 2012). Yet even though most of these reasons are well known, the most frequently asked question for any journal is ‘what’s your impact factor?’

The consequences of such a narrow view of research assessment have been discussed many times (Vale, 2012; Vosshall, 2012). There is intense competition for publication in high-impact-factor journals, frequently resulting in multiple rounds of review and revision; and if the manuscript is ultimately rejected, the whole depressing cycle is often repeated at a new journal. The resultant delays in the communication of new findings hinder scientific progress and waste limited resources. The focus on publication in a high-impact-factor journal as the prize also distracts attention from other important responsibilities of researchers—such as teaching, mentoring and a host of other activities (including the review of manuscripts for journals!). For the sake of science, the emphasis needs to change.

Anecdotally, we as scientists and editors hear time and again from junior and senior colleagues alike that publication in high-impact-factor journals is essential for career advancement. However, deans and heads of departments send out a different message, saying that letters of recommendation hold more sway than impact factors in promotion and tenure decisions (Abbott et al, 2010; Zare, 2012). Moreover, some research funders (including the Wellcome Trust and Research Councils UK) now stress that assessments of funding applications should focus on the merits of the work proposed rather than the journals (and therefore their impact factors) in which an applicant has published. Similarly, researchers on the sub-panels assessing the quality of research in higher education institutions in the UK as part of the Research Excellence Framework (REF) have been told: ‘No sub-panel will make any use of journal impact factors, rankings, lists or the perceived standing of publishers in assessing the quality of research outputs’. However, there is evidence that some universities are making use of journal impact factors when selecting the papers that will be included in their submission to the REF (Rohn, 2012). And, it remains sadly true that at many institutions in countries where the internal resources may be inadequate to give proper consideration to expert letters and thoroughly review a candidate’s published work, the impact factor remains a convenient crutch on which to base an imperfect evaluation of merit.

There are, however, early signs of an encouraging shift in focus from the journal in which a finding is published to the work itself, with this shift being supported by the availability of metrics at the level of individual articles for many journals. PLOS have been pioneers in this area and, since 2009, have been providing a rich array of metrics on every article published. Using these approaches, assessment can be further extended to a broader array of research outputs, via services that support the deposition of outputs other than full articles, such as Dryad (for datasets), Figshare (for the results of individual experiments, figures, datasets), and Slideshare (for presentations). The emergence of new services, such as Altmetric, Impact Story and Plum Analytics, which aggregate media coverage, citation numbers, social web metrics and so on of individual research outputs, will also provide authors with a more complete picture of the impact of their research.

The changes that are slowly taking place, and which are being facilitated by new technology and tools, lend support to the view that it is time for the research community to reclaim ownership of research evaluation (Vale, 2012). The Declaration on Research Assessment identifies some steps that can now be taken. Recommendations are proposed for all of the key constituencies involved–researchers, publishers, institutions and funders–because it will take commitment and persistence across these groups if we are to reform current practices.

At *eLife*, we strongly support the improvement of research assessment, and the shift from journal-based metrics to an array of article (and other output) metrics and indicators. If and when *eLife* is awarded an impact factor, we will not promote this metric. Instead, we will continue to support a vision for research assessment that relies on a range of transparent evidence–qualitative as well as quantitative–about the specific impacts and outcomes of a collection of relevant research outputs. In this way, the concept of research impact can be expanded and enriched rather than reduced to a single number or a journal name.

With less (or ideally no) involvement of impact factors in research assessment, we believe that research communication will undergo substantial improvement. Journals can focus on scientific integrity and quality, and promote the values and services that they offer, supported by appropriate metrics as evidence of their performance. Authors can choose their preferred venue based on service, cost and reputation in their field. All constituencies will then benefit from a deeper understanding of the significance and influence of our collective investment in research, and ultimately a more effective system of research communication.
